# A “Ballpark” Assessment of Social Distancing Efficiency in the Early Stages of the COVID-19 Pandemic

**DOI:** 10.3390/ijerph19031852

**Published:** 2022-02-07

**Authors:** Taejong Kim, Hyosun Kim

**Affiliations:** 1 KDI School of Public Policy and Management, Sejong 30149, Korea; tjkim@kdischool.ac.kr; 2 Chung-Ang Business School, Chung-Ang University, Seoul 06974, Korea

**Keywords:** COVID-19, social distancing, welfare loss, pandemics, efficiency

## Abstract

This paper presents an efficiency assessment of social distancing as an internationally adopted measure to tackle the COVID-19 pandemic in 2020. The simple framework adopted for the assessment accounts for two kinds of costs that a society may bear in a pandemic. The first is welfare loss due to infection and its consequences, and the second is welfare loss resulting from a slowdown in economic transactions. We call the first infection costs, and the second economic costs, for convenience in the paper. Efficient social distancing should minimize the sum of these costs. Infection costs are likely to decrease with social distancing at a decreasing rate as intensified social distancing eases pressure on scarce resources for intensive care. Economic costs on the other hand are likely to increase at an increasing rate as extreme slowdown in economic life may entail job losses and business failures. The resulting U-shaped total costs curve implies parity between infection costs and economic costs as a necessary condition for efficiency. In a simplified implementation of the framework, we approximate infection costs by the value of (statistical) lives lost, and economic costs by the gap between the actual gross domestic product (GDP) in 2020 and the potential GDP as predicted by the within-country growth trend during the preceding decade. The results for 158 countries suggest that the global community perhaps reacted with overly strict social distancing measures. The results for the subgroup of high-income countries, however, suggest that these countries were more successful in maintaining the parity between infection and economic costs.

## 1. Introduction

The COVID-19 pandemic has claimed more than 5.2 million lives as of the end of November 2021. Economic consequences have also been devastating. The global gross domestic product (GDP) recorded a 3.5% contraction in 2020, and while economic activities have regained some momentum in 2021, the projection for the global GDP in 2021 remains below the pre-pandemic levels across the regions [1]. A key component in global responses to the pandemic has been various measures of social distancing [2,3,4,5]. While the remarkably fast development and deployment of vaccines has significantly enhanced the ability of the global community to control the spread of the virus, viral mutations necessitate continued practice of social distancing in emergency situations, even in regions with already high vaccination rates. During the first year of the pandemic, social distancing was the mainstay in public health responses to fight the viral spread across the world, and it remains such in a large swathe of developing countries where vaccine supplies are still slow in arriving. 

**Figure 1:**
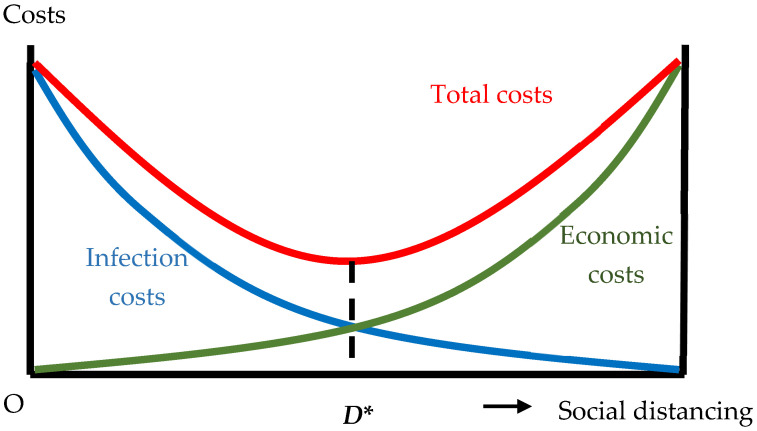
Infection costs, economic costs, and efficient social distancing. (Source: the authors’ own conceptualization).

This paper presents a simple and practical conceptual framework for assessing efficiency in the implementation of social distancing across the countries. The framework recognizes that efficient social distancing should minimize the sum of two distinct costs a society may bear in a pandemic: first, costs due to infection by the virus (infection costs), and second, costs due to the potential slowdown in economic transactions (economic costs). Social distancing will reduce infection costs, while raising economic costs. Figure 1 provides a visual representation of the framework.

Infection costs are likely to decrease with social distancing at a decreasing rate as intensified social distancing eases pressure on limited resources for intensive care [5]. Due to the capacity ceiling in the public healthcare system for intensive care, modest social distancing may save a lot of lives, whereas public health benefits from further restrictions in mobility are likely to be smaller. Economic costs on the other hand are likely to increase at an increasing rate as the extreme slowdown in economic life entails job losses and business failures [6,7,8]. As social distancing intensifies, the reduction in business customs will mount. Businesses will be forced to lay-off employees, and even to close down, in case social distancing is strengthened beyond a point, with discrete jumps in implied welfare loss. The resulting U-shaped total costs curve implies parity between infection costs and economic costs as a necessary condition for efficiency. In Figure 1, efficiency is attained at $D^{*}$, where infection costs and economic costs match each other, minimizing total costs. 

Rigorous measurement of infection and economic costs is not an easy task [9]. In this paper, we attempt a simplified operationalization by approximating infection costs by the value of (statistical) lives lost, and economic costs by the gap between the actual gross domestic product (GDP) in 2020 and the potential GDP as predicted by the within-country growth trend during the preceding decade. These approximations are both likely to understate the true costs, as they leave out obvious items: infection costs should also include costs for treatment for the infected [10], value of labor lost while the infected go through treatment and recovery [11], and long-term adverse health effects of infection known as “long COVID” [12], whereas economic costs should include welfare loss due to negligence in the care of other diseases [13], loss in psychological wellbeing due to imposed loneliness [14], and disruptions in human capital investment, among other things [15].

Our motivation for choosing the simplifying approximations is chiefly practical, as we have no clear methods to estimate the omitted items in a credible and comparable manner across the countries in the world. In the case of the infection costs, however, we believe that our approximation is likely to capture more or less satisfactorily the true extent of the costs, as the value of lives lost will dwarf the other items. We concede that the extent of the underestimation is likely to be more substantial in the approximation we propose for the economic costs. We will discuss the implication of this observation in the discussion of the results.

We followed the recommendation by Viscusi and Masterman [16] to estimate 2020 values of a statistical life (VSL) in different countries, and multiplied these by the number of cumulative deaths due to COVID-19 by the end of year 2020 provided by the World Health Organization (WHO) to estimate country-by-country infection costs. We sourced GDP data for 2020 and the preceding decade from the World Bank.

We have already noted that infection costs should be equal to economic costs, if social distancing is to be efficient and total costs are minimized. Thus, the ratio of infection costs over economic costs should be equal to 1 with efficient social distancing. The value of the ratio over 1 means that infection costs outweigh economic costs, and that social distancing is insufficient in view of efficiency. In the context of Figure 1, social distancing is practiced on the left-hand side of the optimal point $D^{*}$. The value of the ratio below 1 means the opposite: economic costs from social distancing outweigh infection costs, and social distancing is overly strict on the right-hand side of the optimal point $D^{*}$.

The actual range of the ratio of infection costs over economic costs is widely spread on both sides of the critical value of 1. For our sample of 158 countries, both the mean and the median of the ratios are significantly below 1, suggesting that the vast majority of countries in the global community practiced overly restrictive social distancing. For 34 countries in the sample with per capita income over USD 20,000, however, we cannot reject the null hypothesis that the mean or the median of the ratio is equal to 1.

The rest of the paper proceeds as follows. Section 2 will discuss approximations we used to estimate both infection costs and economic costs. Section 3 will present results from the comparison of infection and economic costs across the countries in the sample, including results from statistical tests. Section 4 will discuss the findings and their implications and present some concluding remarks. 

## 2. Estimation of Infection Costs and Economic Costs

Infection costs comprise costs for medical treatment, value of labor lost by the infected during treatment and recovery, reduction in welfare due to long COVID, and most importantly, value of lives lost. Our “ballpark” estimation focuses on the value of lives lost, ignoring the remaining items. This decision was chiefly forced by the difficulty in estimation for a large number of countries, but may be justified in that the value of lives lost most probably dominates the remaining items by an order of magnitude.

To measure the value of lives lost to the pandemic, we rely on the concept of the value of a statistical life (VSL). The VSL is a measure of life’s value derived from the tradeoff rate between fatality risk and money, often observed through choices in product and labor market contexts [17]. Since the 1980s, the VSL has played an increasing role in cost–benefit analyses for regulatory changes affecting mortality risks in the US and other countries. As most estimates of the VSL are concentrated in a relatively small number of mostly high-income countries, mortality valuation in a global context has to estimate the VSL figures for countries through extrapolation from a base country [18]. The extrapolation relies on the following formula:

(1) $${\mathrm{VSL}}_{\mathrm{target}}={\mathrm{VSL}}_{\mathrm{base}}*{\left({\mathrm{Income}}_{\mathrm{target}}/{\mathrm{Income}}_{\mathrm{base}}\right)}^{\mathrm{elasticity}}$$

In the equation, elasticity is a positive parameter capturing the empirical pattern among existing VSL estimates, showing higher VSL values for more affluent societies. Following the recommendation by Viscusi and Masterman [16], we used 2015 US as the baseline, with the VSL estimated to be USD 9.6 million and the base income of USD 55,980. Elasticity was assumed to be 1.0 for countries with a per capita income of less than USD 8809 and 0.85 for countries with a higher income.

Estimating economic costs poses an even greater challenge. We estimated economic costs through the difference between the actual 2020 GDP and the predicted 2020 GDP estimated using the average annual growth rate during the previous decade. We are aware that this approximation clearly understates the true value, as it ignores welfare loss due to negligence in the care of other diseases, loss in psychological wellbeing due to imposed isolation, and adverse consequences for disruption in the investment for human capital. While we lack a credible method to estimate the value of the omitted items across a large number of countries, country case studies indicate that the welfare reduction implied by these items is indeed substantial [10,11,12,13,14,15]. This realization is the main reason why we think of our assessment as a “ballpark” exercise. We will discuss the implication of the relatively more severe underestimation of economic costs versus infection costs later, and note briefly here that a more accurate measurement of both the costs should strengthen our case that social distancing was overly restrictive during the first year of the pandemic.

## 3. Results

Using data on GDP and COVID-19 deaths sourced, respectively, from the World Bank and the World Health Organization, we have calculated infection costs and economic costs per capita. The summary statistics for these and other related variables are presented in Table 1. From the table, we note that both the mean and, in particular, the median are larger for economic costs than their counterparts for the infection costs.

In Table 2, we present the costs and related data for six selected countries, with the same data covering all the countries in the sample provided in the Appendix A. COVID-19 deaths are the number of cumulative deaths by the end of year 2020, and VSL represents our estimates of the VSL based on extrapolation from the 2015 US baseline. Infection costs in the third column were found by multiplying the number of deaths by VSL. Note that the figures in the table are not normalized for population. Infection costs in countries on the top three rows (China, South Korea, and Australia) were relatively small, measured in billions, mainly reflecting fewer deaths registered in these countries. Infection costs were much higher for Germany, the US, and Belgium, as shown in the table, and in the US in particular, the infection costs were beyond 3 trillion dollars. Economic costs were also substantial, and in the countries in the top three rows, they were larger than the infection costs. In the US and Belgium, we see that while economic costs were large, they were smaller than even the larger infection costs these two countries incurred in 2020. China is notable with the economic costs of over 1 trillion dollars, much larger than their infection costs. Reflecting these cross-country variations, the last column reports a fairly wide range of values for the ratio of infection costs over economic costs. For the six countries in consideration, the ratio varies from 0.0057 for China (overly strict social distancing) to 3.3314 for Belgium (overly lax social distancing).

**Figure 2:**
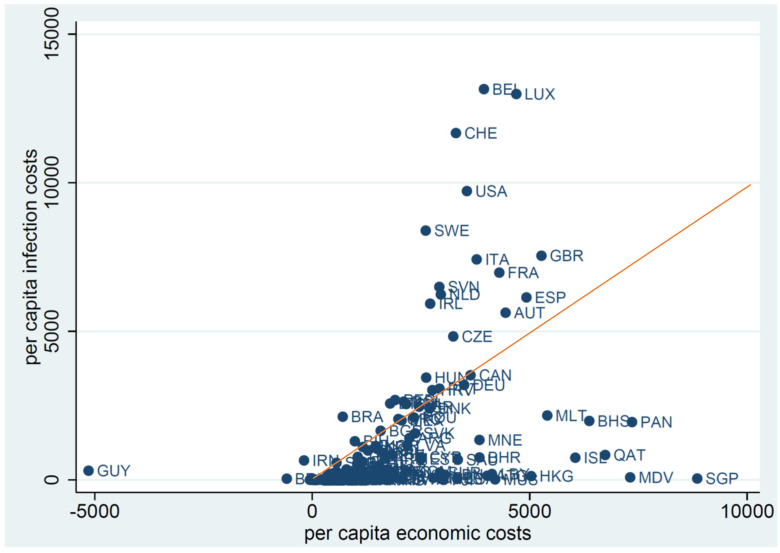
Economic costs versus infection costs: international comparison (in USD).

Figure 2 presents a scatterplot juxtaposing economic costs against infection costs in the international comparison. Each dot in the plot represents one of 158 countries for which we have been able to estimate these costs. The red straight line superimposed on the plot is the 45-degree line, signifying parity between economic costs and infection costs. The countries above the line suffered infection costs higher than economic costs. Had they been able to be stricter in social distancing, they should have been able to cope with the pandemic with lower total costs. The vast number of countries located below the line incurred economic costs larger than infection costs, meaning that they could have relaxed their measures of social distancing and reduced the total costs.

**Figure 3:**
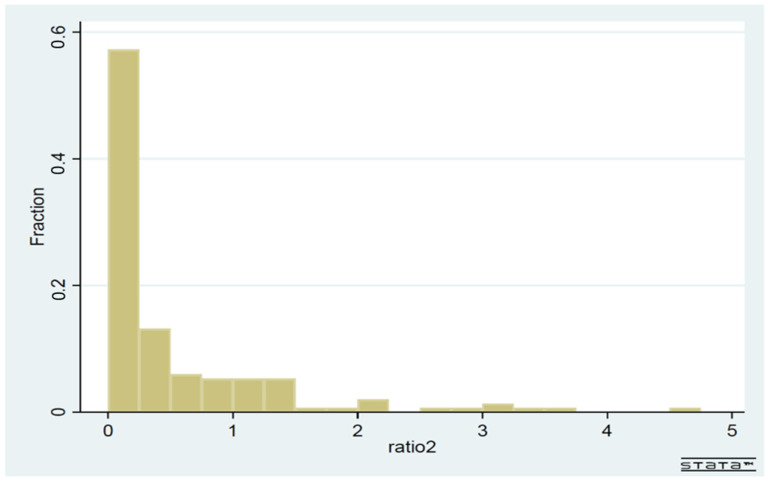
The frequency distribution of the ratio infection costs/economic costs: whole sample (158 countries).

**Figure 4:**
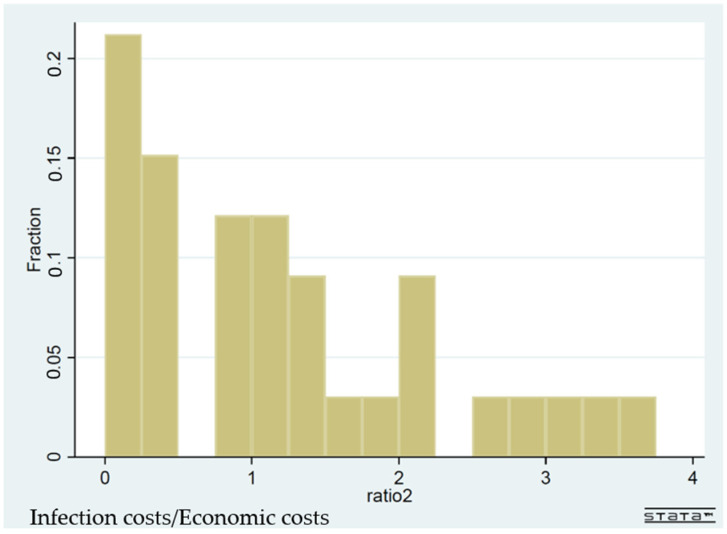
The frequency distribution of the ratio infection costs/economic costs: high-income countries (with per capita GDP over USD 20,000; 34 countries).

Figure 3 and Figure 4 are histograms, showing the frequency distributions of the ratio of infection costs over economic costs, for the whole sample (Figure 3) and for the high-income subsample (Figure 4), respectively. The high-income subsample has 34 countries with a per capita GDP over USD 20,000. Figure 3 vividly demonstrates that the global community in general has overreacted to the pandemic scare in terms of social distancing. In more than 50% of countries, the infection costs/economic costs ratio was under 0.25. In 130 countries out of 158, the same ratio was below 1. In the framework of Figure 1, all these countries’ practice of social distancing was to the right-hand side of $D^{*}$. In Figure 4, the frequency distribution is more closely concentrated around the value of 1 for high-income countries, even though there is no bunching around the value of 1.

We may formally test how successful countries are in efficient practice of social distancing. The variable used in the one-sample *t*-test is the ratio of infection costs over economic costs. If the ratio is at 1 or near 1, we may decide the country was efficient in social distancing in the sense of minimizing the sum of infection and economic costs. Values of the ratio far away from 1 signify failure of efficient social distancing, either overly strict (the ratio much lower than one) or overly lax (the ratio much larger than one). Therefore, we present the null and alternative hypotheses as follows:

$H_{0}$: The mean of the ratio of infection costs over economic costs is equal to 1 (efficient social distancing).

$H_{A}$: The mean of the ratio is not equal to 1 (inefficiency in social distancing).

See Table 3 for detailed results from the one-sample *t*-tests. At conventional levels of significance of either 5% or 1%, we strongly reject the null hypothesis. As a matter of fact, the *p*-value from the test is less than 0.0001. The results from the one-sample *t*-test for the high-income subsample are also presented in Table 3, and do not reject the null hypothesis that the mean ratio is one at conventional levels of significance.

## 4. Discussion and Concluding Remarks

We have applied a simple framework with fairly light data requirements and assessed efficiency in the global practice of social distancing, and found that the intensity in social distancing was overly strict in a majority of countries during the first year of the pandemic. We used the annual timeframe to compare infection and economic costs. In countries where GDP estimates are expediently made available at a higher frequency, it might be possible to put the paper’s framework to a practical, “ballpark”-style assessment of the social distancing practice, for instance on a quarterly basis. 

An ideal and more rigorous assessment of efficiency in social distancing as a response to the COVID-19 pandemic would require a set of elaborate models and a range of reliable data. For instance, Thunström and others [19] used the SIR (Susceptible Infectious Recovered) epidemiological model to estimate expected numbers of deaths and used macroeconomic forecasts from the global consulting firm McKinsey under different scenarios of social distancing to “flatten out” the curve in the initial phase of the pandemic. An obvious drawback of rigorous approaches for a practical global assessment is the unavailability of parameter estimates and other data required for a large number of countries. Striking the appropriate balance between saving lives and keeping the economy afloat is an urgent challenge across the world. Our hope is that the simple conceptual framework and the economy in data requirements in our “ballpark” efficiency assessment might provide a practically useful data point for desperate policymakers in the developing world.

For the whole sample, it was obvious that the vast majority of countries in the world were erring on the overly strict side of the balance, that is, on the right-hand side of the optimal distancing level, $D^{*}$, in Figure 1. We have already noted that both infection costs and economic costs are likely to be underestimated in our approximations. In the ratio of infection costs over economic costs, the extent of underestimation is most probably larger for the denominator, that is, the economic costs. This implies that in the absence of measurement errors, the distribution of the values of the ratio of infection costs over economic costs should tilt further to the left, rendering the ratios for countries even further away from unity and closer to zero. 

Assuming no measurement error, parity between infection costs and economic costs should be desired in the normative sense, as a society struggles to cope with the pandemic at the lowest possible costs. We surmise at the same time that there might also be a positive tendency for the ratio to converge to one over time. This would be the case, for instance, if the political processes in a given society are successful in incorporating diverse interests and voices among the public in an efficient manner. This observation suggests two natural extensions to the study that this paper reports: When the GDP data become available for 2021, we should be able to check whether countries do migrate closer to the 45-degree line in a chart as in Figure 2. Additionally, we could investigate what political, social, or cultural factors correlate with the distance between their infection costs/economic costs ratios and unity.

## Figures and Tables

**Table 1:** Summary statistics (in USD, 2015 PPP).

Variable	Obs.	Mean	Std. Dev.	Median	Min.	Max.
Infection costs per capita	158	1244	2486	138	0.005	13,150
Economic costs per capita	159	1601	1836	1071	−5114 *	8852
VSL	159	2,411,469	3,325,592	931,999	33,672	1.67 × 10^7^
GDP per capita	159	14,025	19,631	5434	196	107,028

* We counted six countries in our sample where the actual 2020 GDP was higher than the predicted: Iran, Brunei Darussalam, Guyana, Guinea, Central African Republic, and Comoros.

**Table 2:** COVID-19 deaths, VSL, infection costs, and economic costs in 2020 for selected countries.

Country	COVID-19 Deaths	VSL	Infection Costs in Mil. USD (A)	Economic Costs in Mil. USD (B)	Ratio A/B
China	4634	1,399,381	6485	1,134,762	0.0057
South Korea	917	5,428,300	4978	85,729	0.0581
Australia	909	9,615,503	8740	36,833	0.2373
Germany	33,791	7,903,049	267,974	292,941	0.9148
United States	352,001	9,192,503	3,235,770	1,183,774	2.7334
Belgium	19,528	7,833,682	152,976	45,918	3.3314

**Table 3:** Results from one-sample *t*-tests against the null hypothesis of parity between two costs.

Sample	Obs.	Mean	Std. Err.	Std. Dev.	*T*	Lower B. (95% CI)	Upper B. (95% CI)
Whole sample	158	0.4661	0.0680	0.8549	−7.8495	0.3317	0.6004
High-income	34	1.1785	0.1816	1.0591	0.9828	0.8089	1.5480

## Data Availability

We have used the publicly archived datasets for our analysis. The link to the data sources are included in the references.

## Appendix A

**Table A1:** International Comparison of Efficiency in Social Distancing.

Country	Code	IC	EC	IC/EC	Country	Code	IC	EC	IC/EC
**Egypt, Arab Rep.**	EGY	38	8	4.54	**Costa Rica**	CRI	907	1643	0.55
**Switzerland**	CHE	11,673	3303	3.53	**Ecuador**	ECU	614	1156	0.53
**Belgium**	BEL	13,151	3947	3.33	**Latvia**	LVA	1156	2190	0.53
**Sweden**	SWE	8390	2609	3.22	**Jordan**	JOR	206	392	0.53
**Brazil**	BRA	2126	702	3.03	**South Africa**	ZAF	542	1033	0.52
**Luxembourg**	LUX	12,986	4692	2.77	**Paraguay**	PRY	274	557	0.49
**United States**	USA	9720	3556	2.73	**Sao Tome and Principe**	STP	17	35	0.48
**Slovenia**	SVN	6495	2922	2.22	**Finland**	FIN	847	1823	0.46
**Ireland**	IRL	5930	2711	2.19	**Ukraine**	UKR	228	493	0.46
**Netherlands**	NLD	6240	2957	2.11	**Albania**	ALB	353	786	0.45
**Italy**	ITA	7421	3780	1.96	**Belarus**	BLR	172	391	0.44
**France**	FRA	6976	4301	1.62	**Armenia**	ARM	711	1673	0.42
**Czech Republic**	CZE	4829	3242	1.49	**Malta**	MLT	2168	5404	0.40
**Lithuania**	LTU	2571	1791	1.44	**Eswatini**	SWZ	139	347	0.40
**United Kingdom**	GBR	7544	5270	1.43	**Montenegro**	MNE	1344	3845	0.35
**Peru**	PER	2689	1905	1.41	**Guatemala**	GTM	136	402	0.34
**Bosnia and Herzegovina**	BIH	1300	979	1.33	**Belize**	BLZ	370	1127	0.33
**Hungary**	HUN	3442	2619	1.31	**Azerbaijan**	AZE	245	785	0.31
**Austria**	AUT	5626	4445	1.27	**Bahamas, The**	BHS	1984	6370	0.31
**Poland**	POL	2635	2104	1.25	**Georgia**	GEO	471	1522	0.31
**Spain**	ESP	6141	4925	1.25	**Moldova**	MDA	291	941	0.31
**Chile**	CHL	2552	2159	1.18	**Cyprus**	CYP	765	2501	0.31
**Croatia**	HRV	3023	2761	1.09	**Bolivia**	BOL	307	1063	0.29
**Bulgaria**	BGR	1654	1572	1.05	**Estonia**	EST	699	2500	0.28
**Portugal**	PRT	3070	2925	1.05	**Panama**	PAN	1945	7353	0.26
**Greece**	GRC	2048	1980	1.03	**Equatorial Guinea**	GNQ	83	341	0.24
**Serbia**	SRB	574	558	1.03	**Tunisia**	TUN	265	1091	0.24
**Israel**	ISR	2463	2456	1.00	**Australia**	AUS	339	1428	0.24
**Mexico**	MEX	2003	2054	0.98	**Saudi Arabia**	SAU	678	3347	0.20
**Canada**	CAN	3521	3640	0.97	**Kazakhstan**	KAZ	347	1731	0.20
**North Macedonia**	MKD	1107	1162	0.95	**Bahrain**	BHR	755	3844	0.20
**Germany**	DEU	3194	3492	0.91	**Iraq**	IRQ	257	1583	0.16
**Romania**	ROU	2101	2336	0.90	**Honduras**	HND	106	701	0.15
**Denmark**	DNK	2404	2699	0.89	**Trinidad and Tobago**	TTO	262	1833	0.14
**Norway**	NOR	1141	1462	0.78	**Morocco**	MAR	107	789	0.14
**Russian Federation**	RUS	988	1281	0.77	**Gambia, The**	GMB	7	51	0.13
**Turkey**	TUR	774	1046	0.74	**Dominican Republic**	DOM	277	2175	0.13
**Colombia**	COL	1037	1500	0.69	**El Salvador**	SLV	114	912	0.12
**Slovak Republic**	SVK	1566	2366	0.66	**Qatar**	QAT	838	6736	0.12
**Argentina**	ARG	1406	2233	0.63	**West Bank and Gaza**	PSE	108	868	0.12
**Country**	**Code**	**IC**	**EC**	**IC/EC**	**Country**	**Code**	**IC**	**EC**	**IC/EC**
**Iceland**	ISL	747	6049	0.12	**Uganda**	UGA	1	52	0.02
**Cabo Verde**	CPV	112	1240	0.09	**Angola**	AGO	6	370	0.02
**Suriname**	SUR	248	2930	0.08	**Myanmar**	MMR	12	817	0.02
**Uruguay**	URY	148	1873	0.08	**Lesotho**	LSO	4	327	0.01
**Mauritania**	MRT	20	266	0.08	**Mali**	MLI	2	129	0.01
**Jamaica**	JAM	76	1079	0.07	**Zimbabwe**	ZWE	4	352	0.01
**Namibia**	NAM	71	1025	0.07	**Malaysia**	MYS	36	2955	0.01
**Indonesia**	IDN	59	855	0.07	**Togo**	TGO	1	79	0.01
**Korea, Rep.**	KOR	97	1671	0.06	**Maldives**	MDV	85	7310	0.01
**Gabon**	GAB	42	717	0.06	**Botswana**	BWA	22	2081	0.01
**Lebanon**	LBN	174	3045	0.06	**St. Lucia**	LCA	34	3334	0.01
**Sudan**	SDN	11	186	0.06	**Ghana**	GHA	3	320	0.01
**Pakistan**	PAK	9	166	0.05	**Nigeria**	NGA	2	241	0.01
**Kyrgyz Republic**	KGZ	35	679	0.05	**Congo, Dem. Rep.**	COD	0	55	0.01
**Algeria**	DZA	45	893	0.05	**Niger**	NER	0	50	0.01
**Libya**	LBY	198	4131	0.05	**Burkina Faso**	BFA	1	77	0.01
**Bangladesh**	BGD	10	210	0.05	**Sierra Leone**	SLE	1	109	0.01
**Afghanistan**	AFG	5	109	0.05	**Madagascar**	MDG	1	114	0.01
**Senegal**	SEN	6	130	0.05	**Cote d’Ivoire**	CIV	1	239	0.01
**New Zealand**	NZL	37	881	0.04	**Mozambique**	MOZ	0	81	0.01
**India**	IND	36	949	0.04	**China**	CHN	4	786	0.01
**Antigua and Barbuda**	ATG	138	4024	0.03	**Sri Lanka**	LKA	6	1126	0.01
**Nepal**	NPL	9	269	0.03	**Singapore**	SGP	46	8853	0.01
**Cameroon**	CMR	4	135	0.03	**Rwanda**	RWA	1	222	0.00
**Philippines**	PHL	42	1378	0.03	**Mauritius**	MUS	16	4204	0.00
**Haiti**	HTI	4	154	0.03	**Papua New Guinea**	PNG	0	366	0.00
**Nicaragua**	NIC	7	276	0.03	**Thailand**	THA	1	1706	0.00
**Kenya**	KEN	6	243	0.03	**Tanzania**	TZA	0	105	0.00
**Hong Kong SAR, China**	HKG	127	5034	0.03	**Burundi**	BDI	0	11	0.00
**Congo, Rep.**	COG	7	263	0.02	**Vietnam**	VNM	0	268	0.00
**Barbados**	BRB	69	2781	0.02	**Fiji**	FJI	1	3018	0.00
**Ethiopia**	ETH	2	72	0.02	**Mongolia**	MNG	0	1558	0.00
**Uzbekistan**	UZB	8	342	0.02	**Comoros**	COM	3	-54	−0.05
**Zambia**	ZMB	5	239	0.02	**Central African Republic**	CAF	1	−16	−0.05
**Benin**	BEN	1	38	0.02	**Guinea**	GIN	1	−19	−0.05
**Tajikistan**	TJK	2	89	0.02	**Guyana**	GUY	310	−5145	−0.06
**Guinea-Bissau**	GNB	2	115	0.02	**Brunei Darussalam**	BRN	41	−587	−0.07
**Malawi**	MWI	1	48	0.02	**Iran, Islamic Rep.**	IRN	654	−189	−3.46
**Chad**	TCD	1	46	0.02					
**Liberia**	LBR	1	76	0.02					

IC = Infection cost per capita, EC = Economic cost per capita, IC/EC = Infection cost per capita/Economic cost per capita.
